# Role of Adiponectin in Regulating Cytokines and Its Contribution to the Occurrence and Progression of Clinical Mastitis in Holstein Cows

**DOI:** 10.3390/ijms26072898

**Published:** 2025-03-22

**Authors:** Junjun Zhang, Na Chen, Zhen Yang, Yumeng Gao, Bohao Zhang, Jianfu Li, Bin Zhou, Zhixiong Tang, Weitao Dong, Xingxu Zhao, Yong Zhang, Quanwei Zhang

**Affiliations:** 1 College of Life Sciences and Biotechnology, Gansu Agricultural University, Lanzhou 730030, China; 107332202298@st.gsau.edu.cn (J.Z.); cn17352088081@163.com (N.C.); 1073324020324@st.gsau.edu.cn (Z.Y.); 13131850259@163.com (Y.G.); li18419220108@163.com (J.L.); zhoub@st.gsau.edu.cn (B.Z.); zhaoxx@gsau.edu.cn (X.Z.); zhychy@126.com (Y.Z.); 2 Gansu Key Laboratory of Animal Generational Physiology and Reproductive Regulation, Lanzhou 730070, China; zhangbhgs@163.com (B.Z.); tzx18153613730@163.com (Z.T.); d.wt2008@163.com (W.D.); 3 College of Veterinary Medicine, Gansu Agricultural University, Lanzhou 730070, China; 4 College of Animal Science and Technology, Gansu Agricultural University, Lanzhou 730070, China

**Keywords:** clinical mastitis, adiponectin, cytokines, bioinformatics

## Abstract

Cytokines are crucial in various physiological and pathological processes, especially in inflammatory diseases in mammals. However, the comprehensive identification of cytokines and their potential regulatory functions in the mammary glands of Holstein cows suffering from clinical mastitis (CM) remains only partially understood. This study aimed to systematically identify biological processes (BPs) and differentially expressed proteins (DEPs) associated with cytokines and to explore their functions through the analysis of previously obtained data from data-independent acquisition (DIA) proteomics. We confirmed that the dynamic balance between pro- and anti-inflammatory factors is closely associated with dairy mastitis. A total of 4 BPs, comprising 75 upregulated and 16 downregulated DEPs, were identified, particularly in relation to adiponectin (ADIPOQ), which strongly interacts with the other DEPs and participates in peroxisome proliferator-activated receptor (PPAR) and adipocytokine signaling pathways. Immunohistochemical and immunofluorescence staining revealed that ADIPOQ was predominantly localized in the cytoplasm of mammary epithelial cells. Moreover, the expression levels of *ADIPOQ* mRNA and protein in the mammary glands of the CM group were notably reduced compared to those in the healthy group. A potential mechanism of action of ADIPOQ was suggested, with findings indicating that a decrease in ADIPOQ expression could potentially worsen inflammation in CM. These results offer novel insights into cytokines and the regulatory mechanisms of ADIPOQ in Holstein cows with CM which may benefit the prevention and treatment of dairy mastitis.

## 1. Introduction

Bovine mastitis is a localized inflammatory response in the udder tissues of cows that is triggered by various factors [1]. Clinical mastitis (CM), a type of cow mastitis, is characterized by symptoms such as swelling and redness of the udder, elevated body temperature, decreased milk production, and changes in milk consistency [2]. These symptoms directly impact the health of dairy cows and affect the yield and quality of dairy products, leading to significant economic losses for the dairy industry [3]. Over the past few decades, various approaches, such as vaccination, antibiotic therapies, environmental management, and novel treatments, have been used to prevent and treat mastitis in dairy cows [4,5,6]. Additionally, the pathogenesis and regulatory mechanisms of cow mastitis have been extensively investigated. However, CM remains prevalent in large-scale farms. Therefore, future research should focus on developing and applying green, precise, and integrated control strategies to tackle the challenges of dairy mastitis.

Cytokines, a class of small proteins or peptides secreted by functional cells [7], are crucial in signal transmission between cells or regulating cell growth, differentiation, and immune response [8]. Similarly, they play a key role in the pathogenesis, diagnosis, and management of CM [9]. Previous studies have confirmed the critical role of cytokines in immune cells such as monocytes, macrophages, lymphocytes, and natural killer cells, as well as their regulatory mechanisms in the progression, diagnosis, and treatment of CM [10]. For example, pathogen invasion induces immune cells (such as macrophages and neutrophils) in mammary gland tissues to release various cytokines, such as interleukin-1β (IL-1β), IL-6, and tumor necrosis factor-α (TNF-α), which promote the development of the inflammatory response [11]. Furthermore, cytokines attract more immune cells to the invasion sites in mammary gland tissues and enhance local immune responses [12]. In addition, cytokines trigger inflammatory responses and promote tissue repair and regeneration [13]. Cytokines are significant in the prevention and treatment of CM owing to their advantages, as they function as markers for early diagnosis and immunomodulators that enhance the immune response and improve resistance to diseases, as well as improve therapeutic effects in combination with other treatments [14]. However, cytokine detection and application present challenges such as detection complexity, individual differences, and potential side effects. The regulatory mechanisms of cytokines are complex and involve the interaction of multiple signaling pathways, which remain partially understood. Moreover, the mechanisms and roles of cytokines in different inflammation stages are not fully understood in terms of individual differences, complicating precision treatment. Bovine mammary epithelial cells (bMECs) are target cells of pathogen invasion and participate in immune regulation by secreting cytokines [11,15]. Several studies have demonstrated that pro-inflammatory cytokines (such as IL-1α, IL-1β, IL-6, TNF-α, and IL-17A) promote the production and release of inflammatory mediators and aggravate inflammation primarily by activating inflammatory signaling pathways in bMECs [16,17,18]. Anti-inflammatory cytokines (IL-4, IL-10, and transforming growth factor-β) reduce inflammatory responses, regulate cell proliferation and apoptosis, and participate in tissue repair by inhibiting the production of pro-inflammatory cytokines [19,20]. Recent studies have discovered and focused on specific novel cytokines and pathways, such as IL-33/growth stimulation expressed gene 2 (ST2) [21], IL-17A/nuclear factor κB (NF-κB) [22], interferon regulatory factor 5/myeloid differentiation primary response protein 88 [23], and beta-defensin-3 [24]. Additionally, studies have found that certain unique cytokines, such as adiponectin (ADIPOQ) and leptin, play crucial roles in the inflammatory response. ADIPOQ is a protein primarily secreted by adipose tissue with various physiological functions, including regulating glucose and lipid metabolism [25], immune responses, and inflammation [26]. ADIPOQ exerts significant anti-inflammatory effects by modulating the expression of inflammatory cytokines [27]. Our previous studies indicated that some crucial pro-inflammatory cytokines are significantly upregulated during the occurrence and progression of CM in dairy cows [18]. However, current studies focus on the preliminary exploration of cytokine expression patterns and signaling pathways; the dynamic regulatory mechanisms of cytokines in MECs require further investigation. The potential of cytokines as tools for managing dairy health, as an alternative to traditional diagnostic and treatment methods, has yet to be thoroughly evaluated.

In this study, we identified the biological processes (BPs), pathways, and differentially expressed proteins (DEPs) related to cytokines using our previous data-independent acquisition (DIA) proteomic data. Our goal was to further investigate the potential mechanisms underlying these processes and validate them through various molecular biology assays. These findings may aid in discovering new cytokines and their regulatory mechanisms and provide novel potential targets for treating dairy mastitis.

## 2. Results

### 2.1. Expression Pattern Analysis of TNF-α, IL-1β, IL-6, and IL-10 in Bovine Mammary Gland Tissues

**Figure 1:**
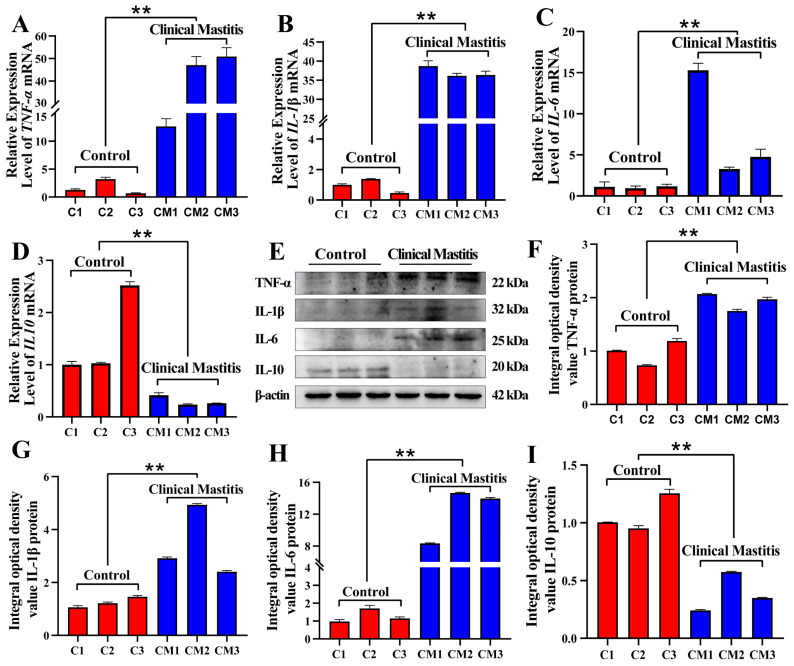
Expression patterns of *TNF-α*, *IL-1β*, *IL-6*, and *IL-10* mRNAs and proteins in bovine mammary gland tissues (MGs) of the control (Con/C) and clinical mastitis (CM) groups. (**A**–**D**): Relative expression levels of *TNF-α*, *IL-1β*, *IL-6*, and *IL-10* mRNAs were evaluated by qRT-PCR assay. (**E**): The immunobands of TNF-α, IL-1β, IL-6, and IL-10 were monitored through Western blotting. (**F**–**I**): The integrated optical density (IOD) of TNF-α, IL-1β, IL-6, and IL-10 bands was digitized using ImageJ 7.0. ** represents *p* < 0.01.

Compared with those in the Con/C group, the relative expression levels of *TNF-α*, *IL-1β*, and *IL-6* mRNAs were notably upregulated in bovine mammary gland tissues in the CM group (*p* < 0.01) (Figure 1A–C). In contrast, the relative expression of *IL-10* mRNA was notably downregulated in the CM group (*p* < 0.01) (Figure 1D). The immunobands of TNF-α, IL-1β, IL-10, and IL-6 in the Con/C and CM groups indicated different expression trends (Figure 1E and Supplementary Figures S1–S5). Similarly, the integrated optical density (IOD) analysis revealed that the relative expression levels of TNF-α, IL-1β, and IL-6 proteins were notably upregulated in the CM group compared to the Con/C group (*p* < 0.01) (Figure 1F–H). In contrast, IL-10 expression was notably downregulated in the CM group (*p* < 0.01) (Figure 1I). The expression patterns of mRNA and protein for TNF-α, IL-1β, IL-10, and IL-6 showed similar trends in both the Con/C and CM groups. These results suggest that the balance between pro-inflammatory and anti-inflammatory factors is disrupted in the pathological process of mastitis, with pro-inflammatory factors predominating in the inflammatory response.

### 2.2. Identification of Candidate BP Terms and DEPs Related to Cytokines in the Bovine Mammary Gland Tissues

**Figure 2:**
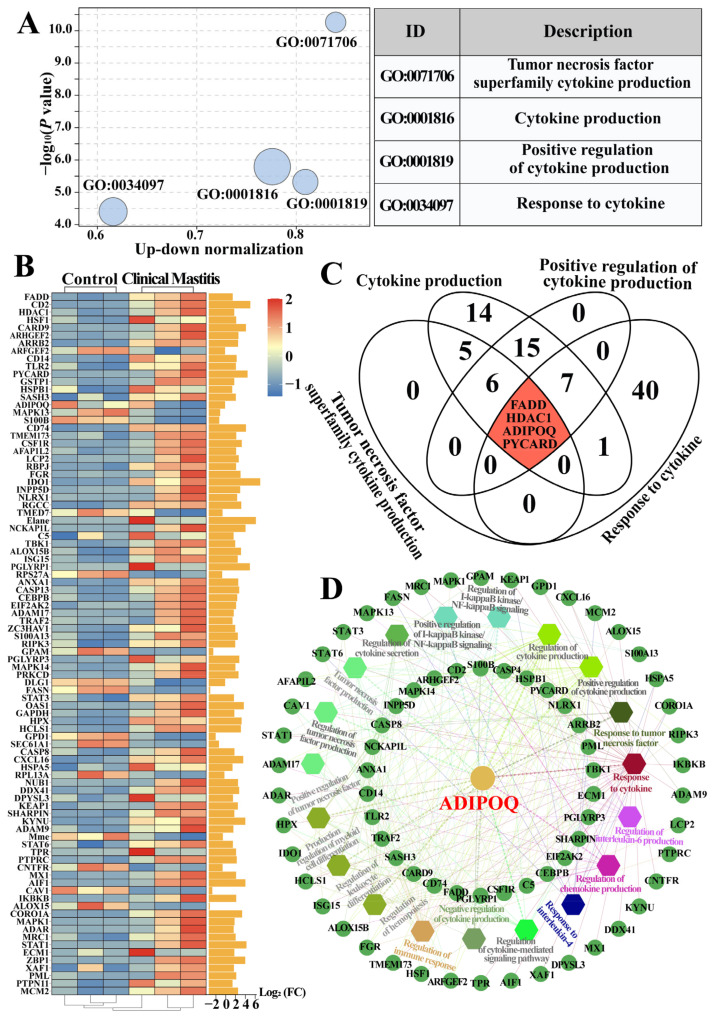
The BPs and DEPs related to cytokines were identified based on GO terms. (**A**): The BP terms associated with cytokines were selected from the DIA proteomic data. (**B**): Dynamic heatmap column diagram of the 91 DEPs. (**C**): Venn diagram of the 4 BP terms and the 91 DEPs. (**D**): Interactive relationship analysis between the 73 DEPs and 19 BPs using ClueGo.

Four significant BPs (*p* < 0.05 and *p.*adjust < 0.05; Table S1), including tumor necrosis factor superfamily cytokine production, cytokine production, positive regulation of cytokine production, and cytokine to response, were screened (Figure 2A). The results yielded 91 DEPs (Table S2). The heatmap showed consistent expression levels of these DEPs in the Con/C group with minimal variation, whereas some of the DEPs exhibited significant variability in the CM group. Moreover, of the DEPs, 75 and 16 were upregulated and downregulated, respectively, with absolute log_2_(FC) values of >0.58 (Figure 2B). The Venn diagram showed that four DEPs, namely FAS-associated death domain protein (FADD), histone deacetylase 1 (HDAC1), ADIPOQ, and apoptosis-associated speck-like protein containing a CARD (PYCARD), were shared among these cytokine-related BPs (Figure 2C). Functional enrichment analysis using the ClueGO plugin revealed a protein–protein interaction (PPI) network comprising 73 DEPs and 19 BP terms, covering 16 cytokine response and secretion–modulation-related processes, particularly TNF (Figure 2D). Notably, some DEPs, such as ADIPOQ and FADD, were linked to multiple BP terms, with ADIPOQ showing the strongest association. These results demonstrate that ADIPOQ plays a pivotal role in cytokine production in the MGs of CM cows.

### 2.3. Identification of Pathways and DEPs Associated with ADIPOQ According to the DIA Data

**Figure 3:**
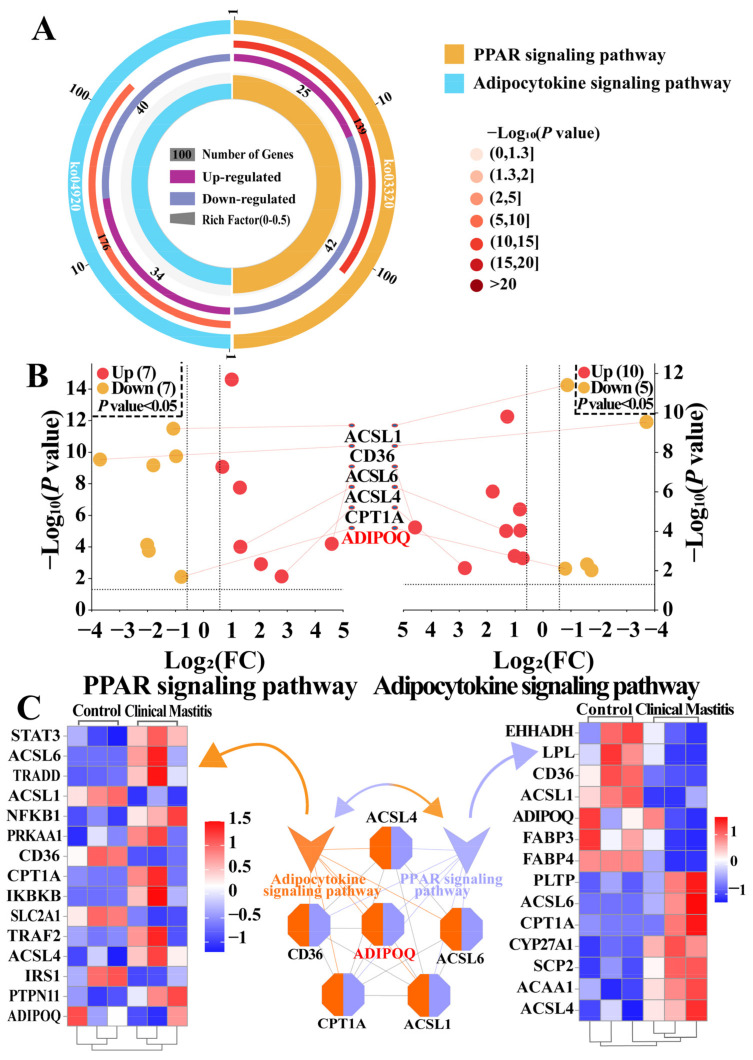
Pathway analysis identifies potential DEPs and pathways related to ADIPOQ between the Con/C and CM groups. (**A**): Candidate DEPs and pathways involving ADIPOQ. (**B**): Dual Volcano plot of two pathways: PPAR (seven downregulated and seven upregulated) and adipocytokine (five downregulated and ten upregulated) signaling pathways. (**C**): PPI and heatmap analysis of the PPAR and adipocytokine signaling pathways involving ADIPOQ.

The enrichment circle plot revealed that ADIPOQ participates in the peroxisome proliferator-activated receptor (PPAR) and adipocytokine signaling pathways (*p* < 0.05 and *p.*adjust < 0.05; Table S3) (Figure 3A). The dual Volcano plot suggested that fourteen DEPs, including seven upregulated and seven downregulated proteins, were identified in the PPAR pathway, while fifteen DEPs, including ten upregulated and five downregulated proteins, were identified in the adipocytokine pathway; six DEPs (Table S4) including acyl-CoA synthetase long chain family member 1 (ACSL1), a cluster of differentiation 36 (CD36), acyl-CoA synthetase long chain family member 6 (ACSL6), acyl-CoA synthetase long chain family member 4 (ACSL4), carnitine palmitoyl transferase 1A (CPT1A), and ADIPOQ were shared in both pathways (Figure 3B). The PPI network results showed that ADIPOQ directly interacted with CD36, CPT1A, and ACSL1, indirectly interacted with ACSL6 and ACSL4, and interacted with the adipocytokine and PPAR signaling pathways. The heatmap showed that some DEPs were notably different between the Con/C and CM groups (Figure 3C). These findings demonstrate that ADIPOQ participates in different cytokine production via the PPAR and adipocytokine pathways, as well as in the onset and progression of CM in cows.

### 2.4. Combined Comparative Screening Based on BPs and Pathways

**Figure 4:**
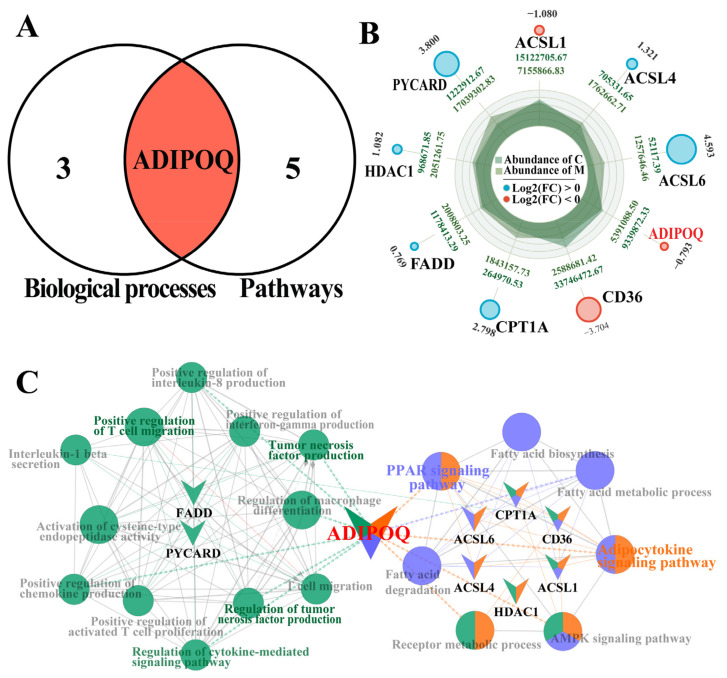
Screening based on the combined analysis of BPs and pathways. (**A**): Venn diagram illustrating the selected BPs and pathways associated with cytokines and their corresponding candidate DEPs. (**B**): Dynamic radar chart of the nine DEPs, including three downregulated and six upregulated proteins. (**C**): Interactive relationship analysis between the shared DEPs and cytokine-related BPs and pathways using ClueGO.

We focused on the common DEPs in BPs and pathways through a combined analysis, identifying four DEPs in the BPs and six in the pathways (Table S5) using a Venn diagram. Among the DEPs shared between BPs and pathways, ADIPOQ was the only overlapping DEP (Figure 4A). The dynamic radar map demonstrated that ACSL1, CD36, and ADIPOQ were downregulated, while ACSL4, ACSL6, CPT1A, FADD, HDAC1, and PYCARD were upregulated (Figure 4B). PPI network analysis revealed that nine DEPs, including FADD, PYCARD, ACSL1, ACSL4, and ADIPOQ, were enriched in six pathways and thirteen BPs. Notably, ADIPOQ is involved in most BPs and pathways, such as the positive regulation of IL-8 production, TNF production, the regulation of macrophage differentiation, and the positive regulation of chemokines, fatty acid metabolism, and receptor metabolism. The pathways associated with ADIPOQ include the regulation of cytokine-mediated signaling pathways, as well as the PPAR, AMP-activated protein kinase (AMPK), and adipocytokine signaling pathways (Figure 4C). These results suggest that ADIPOQ plays a pivotal role as a molecular target in cytokine-mediated responses related to clinical bovine mastitis.

### 2.5. Localization and Expression Analyses of ADIPOQ in Bovine Mammary Gland Tissues

**Figure 5:**
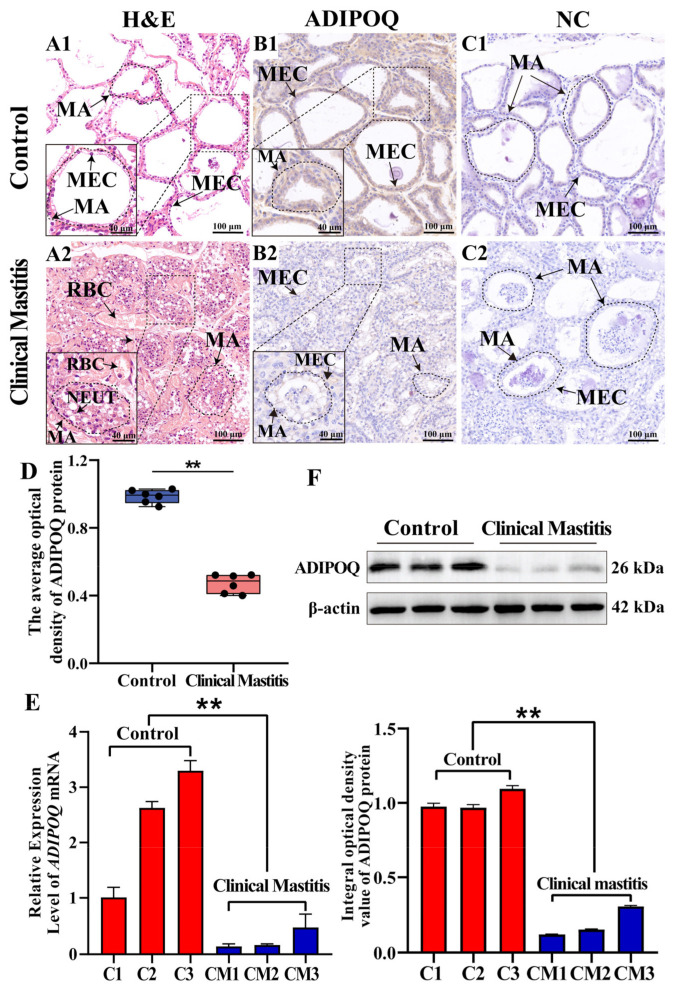
Analysis of ADIPOQ localization and expression in bovine MGs. (**A**): Pathologically examined MGs from the Con/C (**A1**) and CM (**A2**) groups using hematoxylin and eosin staining. (**B**): Localization analysis of ADIPOQ in the MGs in the Con/C (**B1**) and CM (**B2**) groups by immunohistochemistry (IHC). (**C**): Negative control of the MGs in the Con/C (**C1**) and CM (**C2**) groups. (**D**): Analysis of the IOD of ADIPOQ proteins in MGs from the Con/C and CM groups. (**E**,**F**): Relative expression patterns of *ADIPOQ* mRNA (**E**) and protein (**F**) in the MGs of the Con/C and CM groups. MECs: mammary epithelial cells; MA: mammary alveoli; RBC: red blood cell; NEUT: neutrophil; ** represents a significant difference (*p* < 0.01).

Hematoxylin and eosin staining revealed an orderly arrangement of columnar or cuboidal MECs in the intact alveoli of the bovine MGs in the Con/C group. In contrast, epithelial cells detached from MECs, along with predominant neutrophil infiltration in atrophied and deformed alveoli, were observed in the mammary gland tissues of the CM group (Figure 5A). These findings indicate that the structure of mammary gland tissue in cows with clinical mastitis (CM) is disrupted and associated with inflammatory cell infiltration. IHC revealed that ADIPOQ protein was positively expressed in both the Con/C and CM groups (Figure 5B) and was primarily localized in the MEC cytoplasm within the mammary alveoli (MA). The negative control group showed no ADIPOQ staining (Figure 5C). The IOD analysis indicated a significant reduction in ADIPOQ expression in the CM group (*p* < 0.01) compared to the Con/C group (Figure 5D). qRT-PCR and Western blotting confirmed a significant downregulation of *ADIPOQ* mRNA and protein in the CM group compared to the Con/C group (*p* < 0.01, Figure 5E,F and Supplementary Figures S6 and S7). The results suggest that the reduced expression of *ADIPOQ* mRNA and protein positively correlates with the occurrence and progression of CM in dairy cows.

### 2.6. Subcellular Location Analysis of ADIPOQ Proteins in the MGs

**Figure 6:**
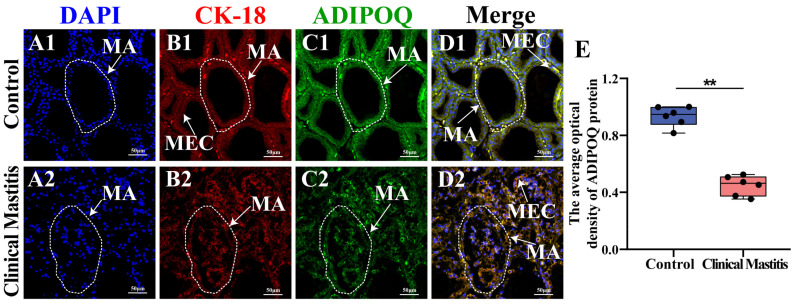
Co-localization analysis of ADIPOQ and CK-18 in MGs. (**A**): The nuclei in the MGs of the Con/C (**A1**) and CM (**A2**) groups were labeled with DAPI (blue). (**B**,**C**): Cellular localization in MGs in the Con/C (**B1**,**C1**) and CM (**B2**,**C2**) groups: CK-18 (red) and ADIPOQ (green). (**D**): Merged CK-18 and ADIPOQ in the MGs in the Con/C (**D1**) and CM (**D2**) groups. (**E**): The IOD of ADIPOQ proteins in the MG tissues in the Con and CM groups. ** represents a significant difference (*p* < 0.01).

Immunofluorescence staining revealed signals for both ADIPOQ and CK18 proteins in MGs of both the Con/C and CM groups (Figure 6). DAPI staining demonstrated that the MEC nuclei were organized in a regular pattern within the MA of the Con/C group. At the same time, they appeared scattered in the CM group (Figure 6A). CK18, a biomarker of epithelial cells, was detected in the MEC cytoplasm in the Con/C and CM groups (Figure 6B). ADIPOQ-positive immunofluorescence signals were observed in the MECs of both groups, with the Con/C group showing stronger signals compared to the CM group (Figure 6C). Co-localization analysis revealed that CK18 and ADIPOQ co-localized within the MEC cytoplasm (Figure 6D). IOD analysis indicated a significant reduction in ADIPOQ expression in the CM group (*p* < 0.01) compared to the Con/C group (Figure 6E). These results suggest that ADIPOQ plays a significant role in MEC function and may influence the emergence and development of CM in dairy cows.

### 2.7. Multiple-Sequence Alignments, Tertiary Structure, and Potential Mechanism Prediction

**Figure 7:**
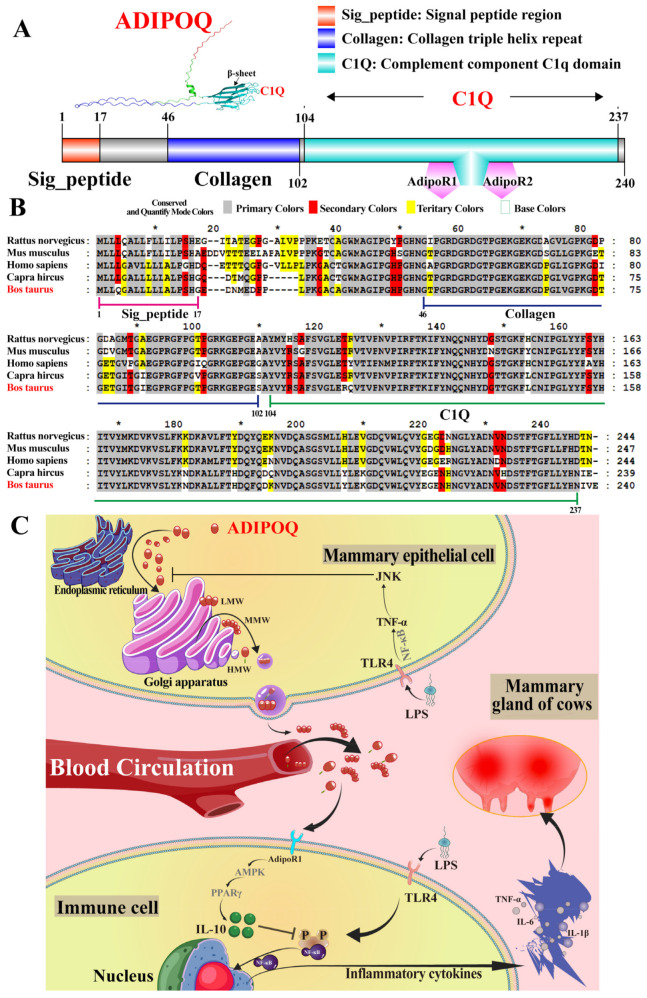
The potential molecular mechanism of ADIPOQ within the MGs of dairy cows affected by CM was revealed. (**A**): The predicted tertiary structure and function of ADIPOQ using AlphaFold 3 and the Illustrator for Biological Sequence software. (**B**): Multiple-sequence alignments of ADIPOQ in different animals using MEGA software. (**C**): The molecular mechanism of ADIPOQ in the MGs was deduced based on pathway analysis.

To predict the potential function of ADIPOQ in the bovine mammary glands in the presence or absence of CM, tertiary structure prediction, multiple-sequence alignments, and a potential mechanism supposition were performed (Figure 7). The IBS results showed that ADIPOQ comprises three regions: the signal peptide region (Sig_peptide, amino acids: 1–17), collagen triple helix repeat (collagen, amino acids: 46–102), and complement component C1q domain (C1Q, amino acids: 104–240) (Figure 7A). Sig_peptide is responsible for guiding synthesized proteins into the endoplasmic reticulum and secreting them from the cell. The Collagen domain, which is enriched with glycine and proline repeats, forms a typical three-stranded helical structure responsible for enhancing protein stability and functionality. The C1q domain, the functional core domain of the ADIPOQ protein, contributes to the binding to Adipoq receptor 1 (R1) and Adipoq receptor 2 (R2), which activate downstream signaling pathways and participate in regulating various physiological functions (Figure 7A). Multiple-sequence alignments showed that ADIPOQ is highly conserved in different mastitis-susceptible species, particularly in cattle and goats, with highly similar amino acid sequences; this suggests that ADIPOQ is highly evolutionarily conserved and may perform similar functions in cattle and goats. However, several amino acid mutations have been observed in cattle and other species. These mutations may lead to changes in protein structure, stability, or receptor-binding ability, thereby affecting the function of ADIPOQ in different species (Figure 7B). The potential mechanism by which ADIPOQ exerts its function in the mammary glands of dairy cows affected by CM was inferred based on the results (Figure 7C). ADIPOQ is bio-synthesized in the endoplasmic reticulum, modified after translation to form a monomer, and assembles in the Golgi apparatus into Trimer (Low-Molecular-Weight Trimer, LMW), six polymers (Medium-Molecular-Weight Hexamer, MMW), or high-molecular-weight polymers (HMW), which are transported and secreted into the extracellular or blood circulatory system by vesicles responsible for regulatory function [28]. When pathogen-associated molecular patterns such as lipopolysaccharide bind to Toll-like receptor 4 on the surface of MEC and immune cell membranes, they activate nuclear NF-κB and promote the expression of inflammatory factors [29,30]. Moreover, ADIPOQ deficiency hinders the binding to the AdipoR1, resulting in the inactivation of the AMPK-PPARγ axis and the failure of the anti-inflammatory mechanism [31,32].

## 3. Discussion

Cytokines are immunomodulatory molecules that participate in local bovine mammary gland inflammatory diseases by mediating inflammation and regulating immune cell function [8,11]. Cytokines play a dual role in clearing pathogens by activating the immune response or causing tissue damage through excessive release [13]. Thus, obtaining a more comprehensive understanding of the roles and regulatory mechanisms of cytokines can contribute to developing effective strategies for preventing, regulating, and treating CM in dairy cows.

The expression levels of several common pro-inflammatory cytokines (IL-6, IL-1β, and TNF-α) and an anti-inflammatory cytokine (IL-10) were assessed in both the Con/C and CM groups (Figure 1). The results demonstrate that the expression levels and dynamic balance of inflammatory and anti-inflammatory factors are crucial in the occurrence, development, and outcomes of CM. However, the systematic identification of cytokines has not been performed in bovine mammary glands with CM. Here, we focused on the BPs, pathways, and DEPs related to cytokines according to our previous DIA Proteomics. A total of 4 BPs comprising 91 DEPs were identified (Figure 2). Previous studies have shown that the excessive expression of some DEPs, such as FADD [33], HDAC1 [34], and PYCARD [35], induces apoptosis and affects the normal functioning of mammary gland cells, which is related to the intensity and duration of the inflammatory response. Specific DEPs, such as caveolin-1, fatty acid synthase, and ADIPOQ, are significant in lipid metabolism and multiple inflammatory diseases [36,37,38] by regulating the expression of pro- or anti-inflammatory cytokines crucial in cytokine production and CM. Future studies should explore the specific mechanisms of these DPEs in dairy mastitis and their potential as therapeutic targets. PPI showed that ADIPOQ had the strongest interaction with the other DEPs. ADIPOQ, a crucial adipocytokine, is secreted by adipose tissue and performs several physiological functions, such as regulating glucose and lipid metabolism and exerting anti-inflammatory effects [39]. ADIPOQ inhibits the production of pro-inflammatory cytokines, such as TNF-α, IL-6, and IL-1β, and enhances that of anti-inflammatory cytokines, including IL-10 [36]. Moreover, ADIPOQ possesses antioxidant properties that help reduce oxidative stress [40]. These findings suggest that ADIPOQ is a significant target for inflammatory diseases, implying its crucial role in dairy mastitis. However, studies on the role of ADIPOQ in dairy mastitis are lacking.

The pathway results suggested that ADIPOQ participates in the PPAR and adipocytokine signaling pathways (Figure 3). These pathways are associated with the occurrence and regulation of CM, and the potential mechanism primarily involves the inhibition of the inflammatory signaling pathway and regulation of immune cell function [41]. For example, activated PPARγ inhibits the activity of inflammatory signaling pathways such as NF-κB and MAPK [42]. PPARγ reduces the production of inflammatory factors by competing with NF-κB for binding sites and inhibiting its activity [31,32]. Leptin induces the production of inflammatory factors such as TNF-α and IL-6, exacerbating inflammation [43]; ADIPOQ has anti-inflammatory properties, inhibiting inflammation by activating the AMPK signaling pathway, regulating fatty acid metabolism, and reducing the synthesis of inflammatory mediators [44]. Moreover, some DEPs, such as CPT1A, CD36, and ACSL1, promote fatty acid oxidation and reduce lipid accumulation and inflammatory factor release [45,46,47]. A combined analysis also confirmed that these DEPs, particularly ADIPOQ, were positively associated with cytokine production and cow CM (Figure 4).

Further investigation through IHC, qRT-PCR, and Western blotting revealed weak ADIPOQ expression in MECs in the CM group. The levels were negatively correlated with CM severity, indicating a potential anti-inflammatory role in cow CM (Figure 5). Co-localization suggested that the decreased ADIPOQ levels in the CM group are related to its reduced anti-inflammatory effects (Figure 6), which could exacerbate mastitis, supporting the conclusion that ADIPOQ mitigates the harmful effects of pathogens on epithelial cells by activating anti-inflammatory pathways [48]. Based on the above results, multiple-sequence alignment, tertiary structure, and potential mechanisms of ADIPOQ were performed and summarized (Figure 7). However, some limitations must be considered, and further validation through assays at both the cellular and animal levels is necessary.

This study illustrates that ADIPOQ directly or indirectly regulates the dynamic balance of cytokines in cows with CM and influences the occurrence and progression of the disease.

## 4. Materials and Methods

### 4.1. Sample Collection

Holstein cows with similar lactation stages were selected from a large-scale and standardized dairy farm in Wuzhong City (Ningxia Hui Autonomous Region, Ningxia, China). They underwent udder health assessment, including redness, swelling, heat, and hardness, as well as somatic cell counts (SCCs), the Lanzhou Mastitis Test (LMT), and veterinary clinical diagnosis, as described previously [49]. Healthy Holstein cows (*n* = 3) without evident udder lesions, with SCC between 7 × 10^4^ and 1 × 10^5^ cells/mL, and negative LMT results were selected as the controls (Con/C). Conversely, Holstein cows (*n* = 3) with evident clinical characteristics, SCC ranging from 13 to 15 × 10^5^ cells/mL, positive LMT results, and milk samples containing water, clots, or blood were classified into the CM group. Fresh udder tissues from the Con/C and CM groups were collected as described. Part of the tissues was cut into 1.5 cm^3^ blocks and fixed in sterile, enzyme-free centrifuge tubes containing 4% paraformaldehyde solution (Solarbio, Beijing, China), while the remaining tissue was cut into 1 cm^3^ blocks and stored in sterile, enzyme-free cryovials at −80 °C for two weeks. The Ethics Committee of Gansu Agricultural University approved this study (Approval No. GSAU-AEW-2018-0128).

### 4.2. RNA Extraction, cDNA Synthesis, and qRT-PCR

Frozen udder tissue samples (20 mg) from both the Con/C and CM groups were homogenized, and total RNA was subsequently isolated using the FastPure RNA Isolation Kit (Vazyme, Nanjing, China). The purity and concentration of RNA were assessed using 1% denaturing formaldehyde agarose gel electrophoresis (Biowest Regular Agarose, Castropol, Spain) and a NanoDrop-8000 spectrophotometer (Thermo Fisher Scientific, Waltham, MA, USA), respectively. cDNA synthesis was performed using a 500 ng RNA sample following a previously outlined method [18]. Primers for bovine *β-actin*, *TNF-α*, *IL-1β*, *IL-10*, *IL-6*, and *ADIPOQ* (Table S6) were designed and synthesized as previously described [49]. qRT-PCR was performed using cDNA as the template, with β-actin as the internal reference gene. The amplification process was performed on the LightCycler^®^ 96 Real-time PCR system with a total reaction volume of 20 μL. The cycling conditions included an initial denaturation at 95 °C for 30 s, followed by 40 cycles of 95 °C for 5 s and 60 °C for 30 s, with a final step at 95 °C for 10 s, 65 °C for 60 s, and 97 °C for 1 s, plus a 37 °C cooling step for 30 s. Each sample was analyzed in triplicate, and relative expression levels of the gene were calculated using the 2^−∆∆Ct^ method.

### 4.3. Western Blotting

Total protein was extracted from 100 mg of tissue samples from cows in the Con/C and CM groups, following the previously described method [49]. A 50 μg aliquot of protein from each sample was utilized to assess the expression levels of TNF-α (1:2000), IL-1β (1:2000), IL-10 (1:3000), IL-6 (1:1000), and ADIPOQ (1:300) (Proteintech, Wuhan, China), with β-actin (with a 1:5000 dilution ratio, Bioss, Beijing, China) serving as the loading control, in the Con/C and CM groups. Immunoblotting was performed as outlined in the previous protocol [18]. The immunobands were analyzed using ImageJ software, version 7.0 (Media Cybernetics Co., Rockville, MD, USA). All immunoblot assays were carried out in triplicate.

### 4.4. Bioinformatic Analysis

DIA proteomic sequencing data from udder tissues of healthy and CM cows (available at https://www.iprox.cn/page/home.html, accessed on 25 February 2024, login ID: IPX0003382000/PXD028100) were used to conduct Gene Ontology (GO) and Kyoto Encyclopedia of Genes and Genome (KEGG) enrichment analyses. We focused on the BPs related to cytokines and DEPs with *p* < 0.05 and *p*.adjust < 0.05, according to the GO enrichment results. ADIPOQ was selected as a significant target DEP, and the pathways and DEPs associated with ADIPOQ were identified, with significance set at *p* < 0.05 and *p*.adjust < 0.05 in the KEGG enrichment results. The R language, version 4.3.3 and OmicShare online platform [49] (http://www.omicshare.com/tools, accessed on 20 March 2024) were used to create Venn diagrams, enrichment circle plots, dynamic heatmap bar charts, and Volcano plots. Additionally, interaction network diagrams for DEPs and GO/KEGG were constructed using the ClueGO plugin in STING (v10.0) and Cytoscape 3.8.2 (Cytoscape Consortium, San Francisco, CA, USA) to analyze the DEPs functions. The tertiary structure of ADIPOQ was analyzed using AlphaFold 3 online software (Google DeepMind, London, UK). The protein sequence structure of ADIPOQ was mapped using the Illustrator for Biological Sequence (IBS 1.0) software (Sun Yat-sen University, Guangzhou, China). Multiple-sequence alignments of ADIPOQ from five animals were performed and imaged using the MEGA 11 (Tempe, AZ, USA) and GeneDoc software, version 2.7 (Pittsburgh, PA, USA). The potential regulatory mechanism of ADIPOQ was predicted according to the pathways and literature reports and illustrated using the Adobe Illustrator 2023 software (Adobe Systems, San Jose, CA, USA) and MedPeer (MedPeer^®^, Beijing, China).

### 4.5. Hematoxylin and Eosin Staining

Fixed udder tissues from both the Con/C and CM groups were embedded in paraffin and sectioned into 5 μm thick slices, following the previously described method [49]. The sections were placed in an oven to bake and deparaffinized using xylene. Afterward, they were rehydrated through a series of graded alcohol solutions and stained with hematoxylin. Differentiation was performed using 1% hydrochloric acid in alcohol, followed by rinsing in running water and counterstaining with eosin, as previously described [49]. After drying, the sections were examined and imaged using an optical microscope (Carl Zeiss, Oberkochen, Germany), with six randomly selected fields analyzed for each sample.

### 4.6. Immunohistochemistry (IHC)

Following deparaffinization and hydration, antigen retrieval was performed on the sections using sodium citrate buffer (Service Bio, Wuhan, China). IHC staining was conducted according to the standard avidin–biotin–peroxidase complex (SABC) kit instructions (BOSTER, Wuhan, China). Rabbit anti-ADIPOQ primary antibody (1:100) was added while using phosphate-buffered saline (PBS) as a negative control. Observations and imaging were performed using a Nikon optical microscope (Nikon, Tokyo, Japan). The IOD of the ADIPOQ protein was quantified and analyzed with ImageJ 7.0 software (Media Cybernetics, Rockville, MD, USA), examining six randomly selected fields per sample.

### 4.7. Immunofluorescence Staining (IF)

After deparaffinization, hydration, antigen retrieval, and 3% H_2_O_2_ incubation, the sections were blocked with 5% (*w*/*v*) donkey serum (Solarbio, Beijing, China) for 40 min. They were then incubated with rabbit anti-CK-18 and rabbit anti-ADIPOQ primary antibodies (1:100; Proteintech, Wuhan, China). Subsequently, the different colors of fluorescent secondary antibodies were applied at a 1:300 dilution, as described previously [49]. Observations and image capture were performed using an Echo-Labs fluorescence microscope and imaging system (Olympus, Tokyo, Japan), with six randomly selected fields analyzed per sample.

### 4.8. Statistical Analysis

Due to the limited sample size (*n* = 3 per group), normality tests were not performed. The experimental data were analyzed using SPSS 23.0 (SPSS Inc., Chicago, IL, USA). Data were expressed as means ± SD using Student’s *t*-test (between groups) and one-way analysis of variance (between multiple groups). Significance was determined at *p* < 0.05. Statistical graphs were generated with GraphPad Prism 8.0.2 (GraphPad Software Inc., San Diego, CA, USA).

## 5. Conclusions

We observed that the expression levels and dynamic equilibrium between pro-inflammatory and anti-inflammatory factors are tightly linked to the onset and progression of CM. Therefore, we identified 91 differentially expressed proteins (DEPs) participating in four cytokine biological processes (BPs) utilizing data from DIA proteomics. We found two pathways and six DEPs closely associated with ADIPOQ from the significantly different pathways. Comprehensive analysis indicates that ADIPOQ is a significant molecular target in the cytokine responses related to clinical bovine mastitis. IHC and IF staining revealed that ADIPOQ was predominantly localized in the MECs of both healthy Holstein dairy cows and those with CM. Importantly, compared to the Con/C, the *ADIPOQ* mRNA and protein levels were significantly reduced in the MGs of the CM group. Moreover, we characterized the potential regulatory mechanisms of ADIPOQ in the MG, demonstrating that ADIPOQ modulates cytokine homeostasis in cows with CM through direct and indirect pathways, thereby influencing the initiation and progression of the disease. These findings provide a basis for understanding the role of ADIPOQ in cytokine regulation in Holstein cows with CM and offer novel insights into improving prevention and treatment strategies for bovine mastitis.

## Data Availability

The data that support the findings of this study are available from the corresponding author upon reasonable request.

## Supplementary Materials

The following supporting information can be downloaded at https://www.mdpi.com/article/10.3390/ijms26072898/s1.
