# Supplementary material for: Role of Adiponectin in Regulating Cytokines and Its Contribution to the Occurrence and Progression of Clinical Mastitis in Holstein Cows
Source: Int J Mol Sci. 2025 Mar 22;26(7):2898. doi: 10.3390/ijms26072898 (PMC11988340; doi:10.3390/ijms26072898)
Supplement: Supplementary file 1 [file ijms-26-02898-s001.zip › Supplementary Materials/Supplementary -- Blue Plus ll ProteinMarker instructions.pdf]

The BEST for Life Science

Blue Plus® II Protein Marker (14-120 kDa)

Cat.No. DM111

Storage at -20ºC for two years

Concentration

about 2 µg/5 µl for each band

Description

Blue Plus® II Protein Marker is composed of eight prestained proteins ranging from 14 to 120 kDa. The proteins of 50 kDa and 120 kDa bands are covalently coupled to orange dye. The protein of 14 kDa band is covalently coupled to yellow dye. The other five bands are covalently coupled to blue dye. This prestained protein marker is designed for monitoring the electrophoresis and membrane transfer.

Highlights

- Five blue bands, two orange bands and one yellow band.
- MW range from 14 to 120 kDa.
- Ready-to-use format, direct load on gels without heating.

Storage buffer

100 mM Tris-HCl (pH 6.8), 5 mM EDTA, 10 mM DTT, 10% Glycerol, 1% SDS, 0.01% phenol red.

Notes

- Mix well before use.
- Use 5 µl/well for mini gel, 10 µl/well for larger gel.

Electrophoresis condition

For BioRad Mini Electrophoresis installation and transfer system, electrophoresis at 200 V for 50 minutes, transfer to membrane at 200 mA for 3 hours.

Blue Plus® II Protein Marker
(14-120 kDa)

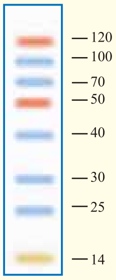

12% Tris-glycine SDS gel (5 µl/well)

FOR RESEARCH USE ONLY

Website www.transgenbiotech.com
E-mail info@transgenbiotech.com
Customer Service +86-400-898-0321
Phone +86-10-57815027
