# Supplementary figures and images for: Role of Adiponectin in Regulating Cytokines and Its Contribution to the Occurrence and Progression of Clinical Mastitis in Holstein Cows
Source: Int J Mol Sci. 2025 Mar 22;26(7):2898. doi: 10.3390/ijms26072898 (PMC11988340; doi:10.3390/ijms26072898)

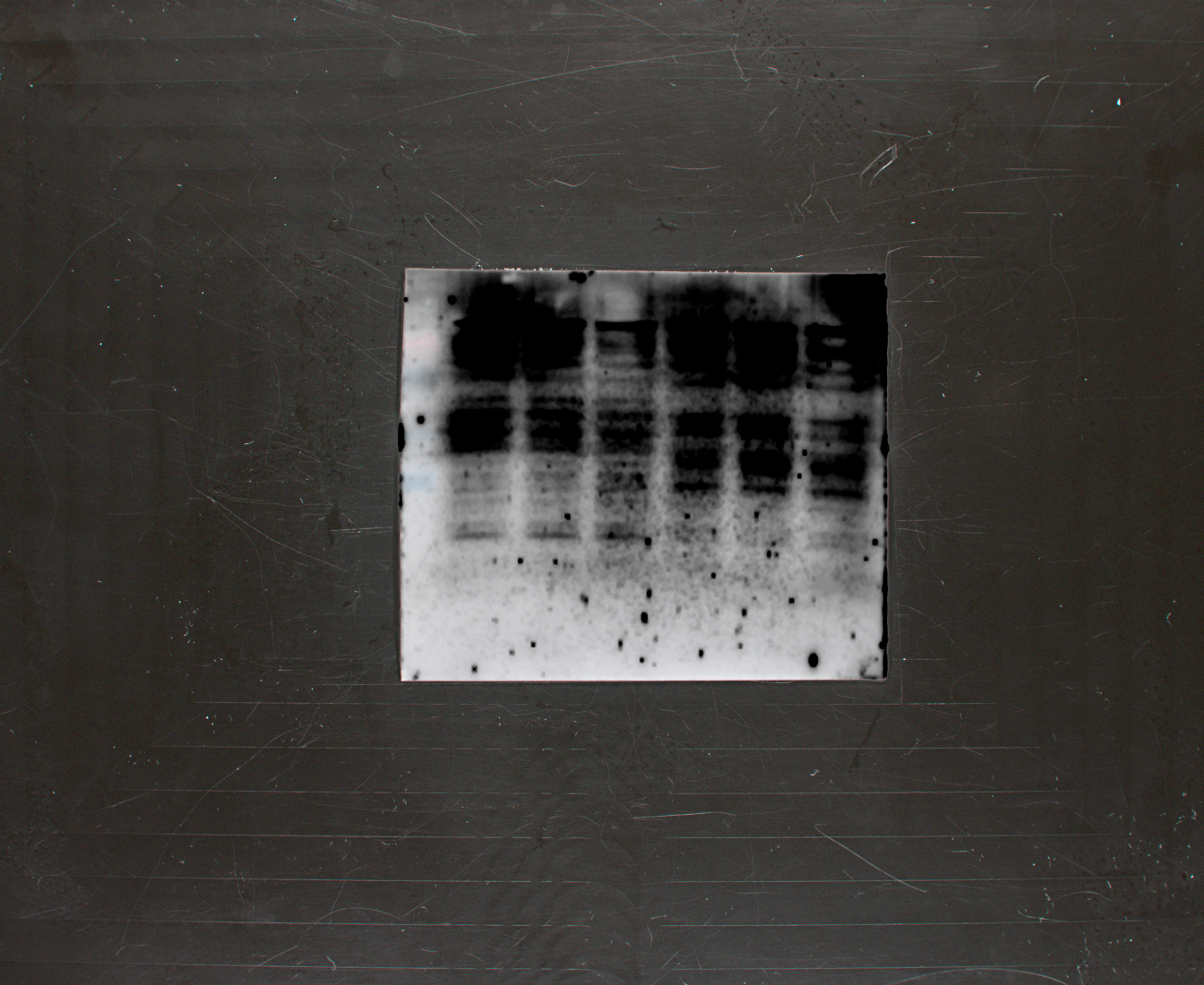

Supplement: Supplementary file 1 [file ijms-26-02898-s001.zip › Supplementary Materials/Figure S1 (TNF-a┴ 22kDa).Tif]

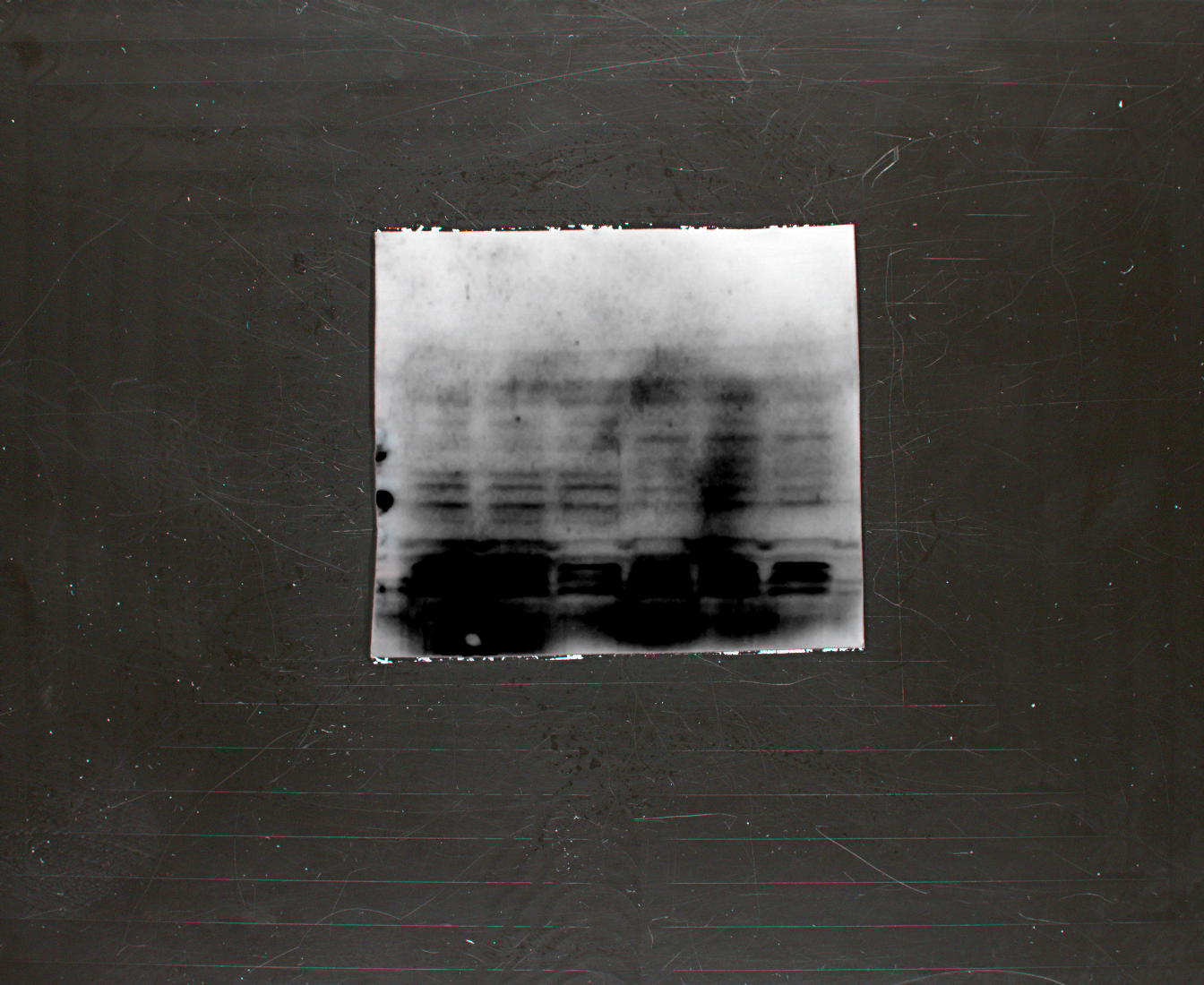

Supplement: Supplementary file 1 [file ijms-26-02898-s001.zip › Supplementary Materials/Figure S2 (IL-1a┬ 32kDa).Tif]

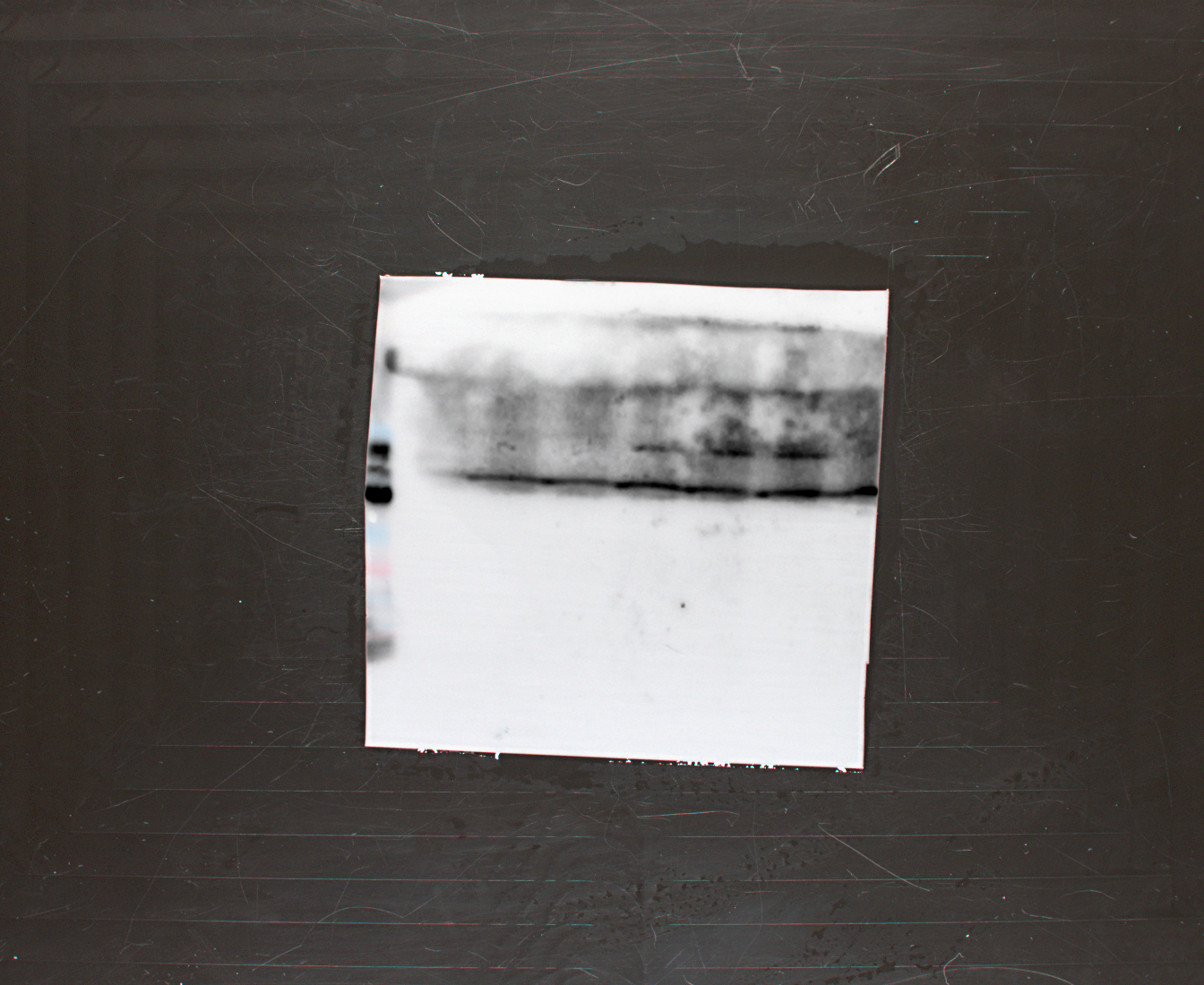

Supplement: Supplementary file 1 [file ijms-26-02898-s001.zip › Supplementary Materials/Figure S3 (IL-6 25kDa).Tif]

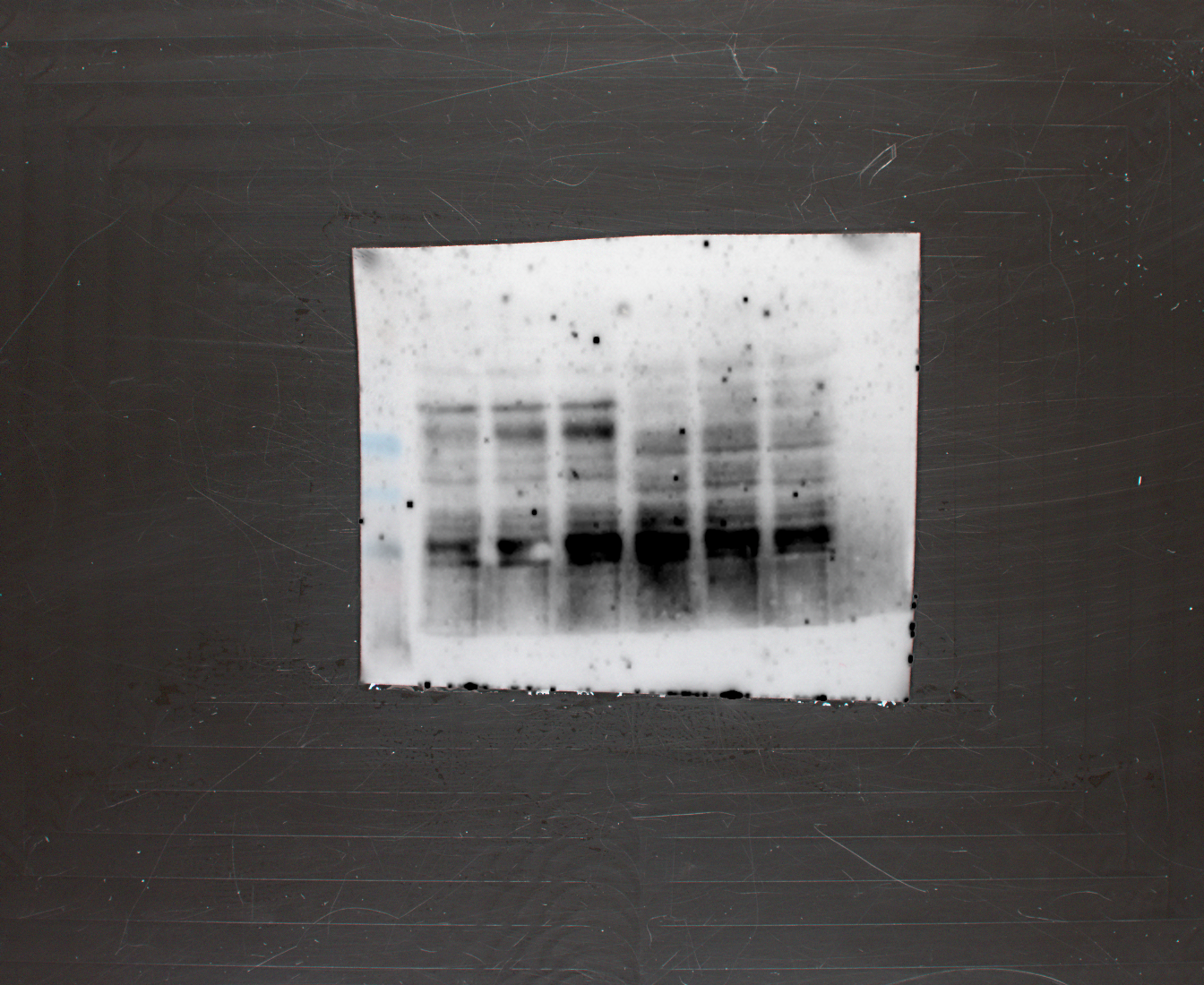

Supplement: Supplementary file 1 [file ijms-26-02898-s001.zip › Supplementary Materials/Figure S4(IL-10 20kDa).Tif]

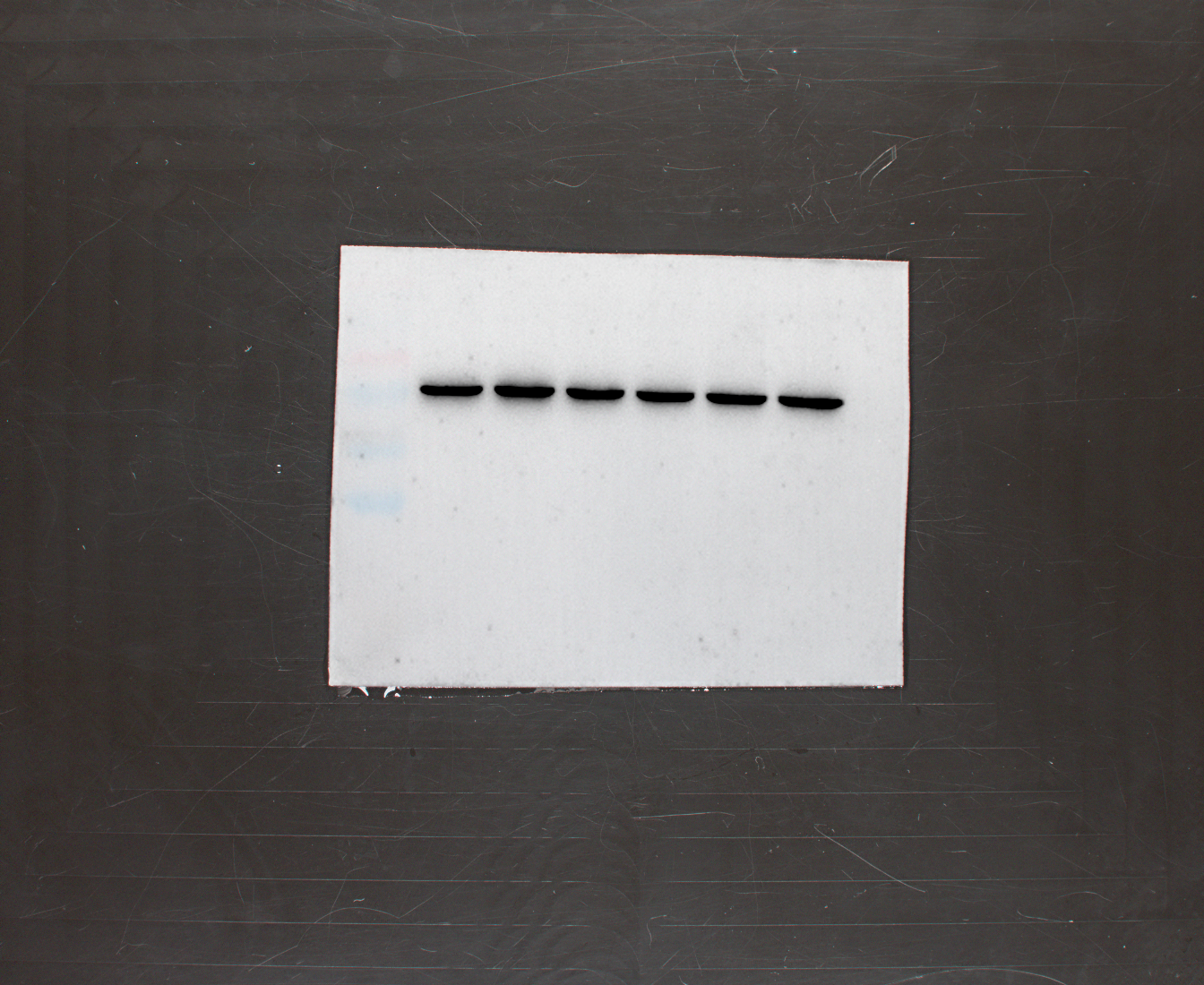

Supplement: Supplementary file 1 [file ijms-26-02898-s001.zip › Supplementary Materials/Figure S5 (a┬-actin 42kDa).Tif]

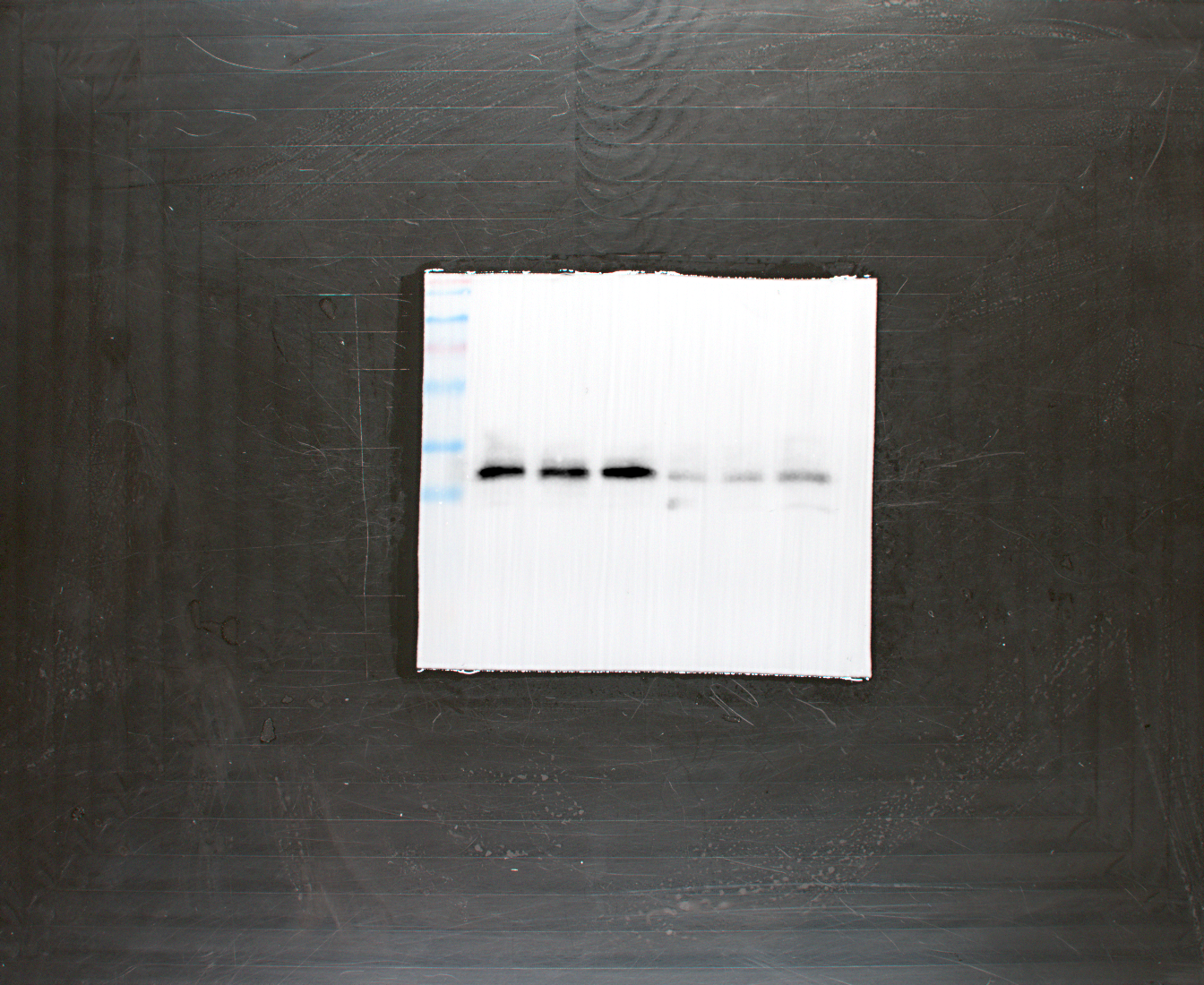

Supplement: Supplementary file 1 [file ijms-26-02898-s001.zip › Supplementary Materials/Figure S6 (ADIPOQ 26kDa).Tif]

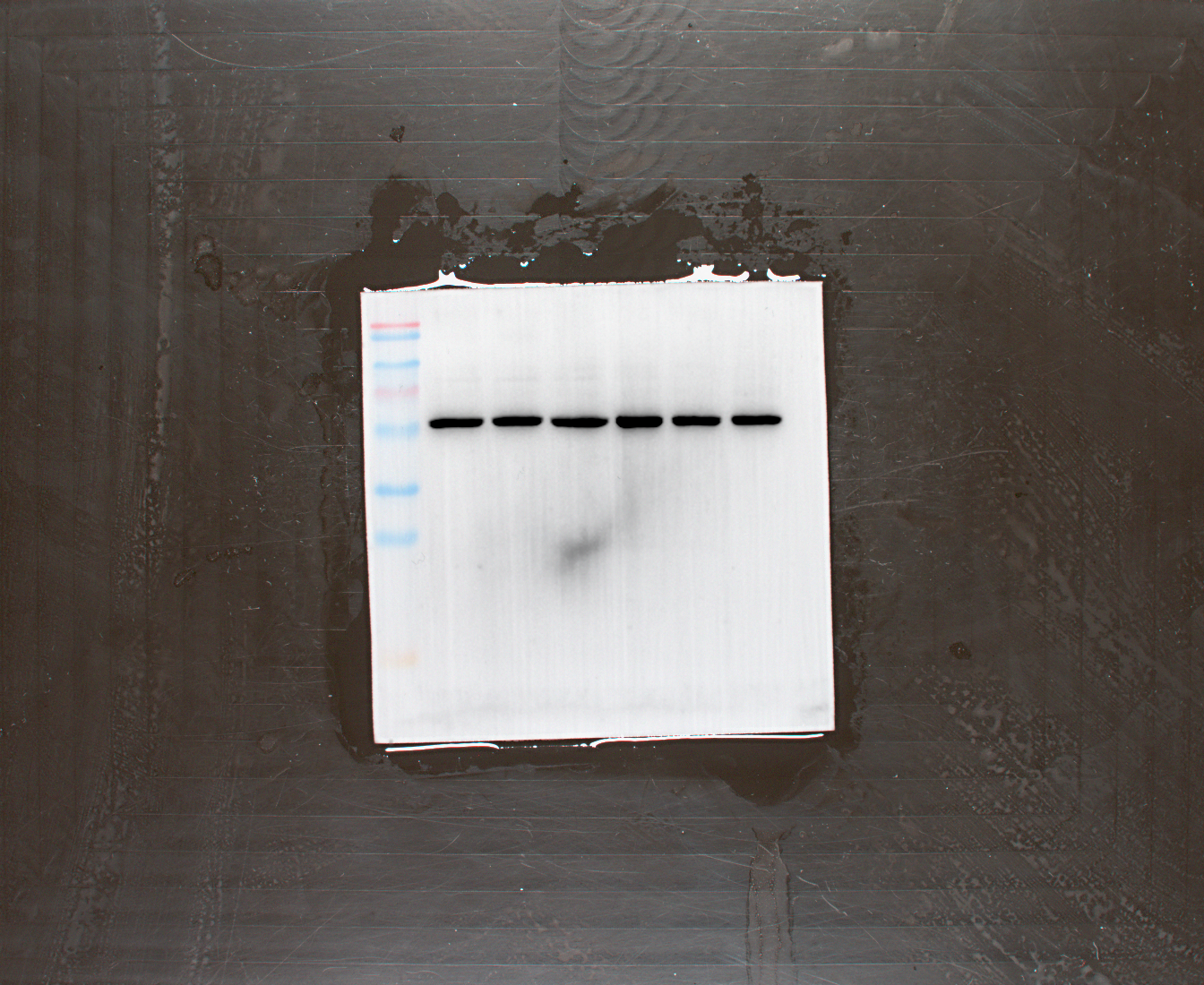

Supplement: Supplementary file 1 [file ijms-26-02898-s001.zip › Supplementary Materials/Figure S7 (a┬-actin 42kDa).Tif]
